# Post-surgical seroreversion in a case of equine cutaneous leishmaniosis by *Leishmania infantum*

**DOI:** 10.1007/s11259-025-11031-0

**Published:** 2026-01-08

**Authors:** Antonio Murillo, María Eugenia Lebrero, Miguel Valdés, Joana Ramos, Sergio Villanueva-Saz, Janine E. Davies, Álex Gómez, Estela Pérez, Cristina Riera, Diana Marteles-Aragüés

**Affiliations:** 1 Hospital de Referencia La Equina, Camino de Martagina Km 1, Manilva, 29692 Málaga, Spain; 2 Clinical Immunology Laboratory, Veterinary Faculty, University of Zaragoza, Calle Miguel Servet, 177, 50013 Zaragoza, Spain; 3 Department of Animal Pathology, Veterinary Faculty, University of Zaragoza, Calle Miguel Servet, 177, 50013 Zaragoza, Spain; 4 Equine Care Group Spain, Calle Santa Engracia, 14-16, 28010 Pozuelo de Alarcón, Spain; 5 Instituto Agroalimentario de Aragón-IA2 (Universidad de Zaragoza-CITA), Calle Miguel Servet, 177, 50013 Zaragoza, Spain; 6 Departament de Biologia, Salut I Medi Ambient, Facultat de Farmacia, Universitat de Barcelona, Av. De Joan XXIII, 08028 Barcelona, Spain

**Keywords:** Dermatology, Granulomatous dermatitis, Horse, *Leishmania infantum*, Serology, Spain

## Abstract

Equine leishmaniosis, caused by *Leishmania infantum* and transmitted by infected sand flies, has been increasingly reported in Europe, although clinical and immunological data remain scarce. We describe a 10-year-old mare from southern Spain presenting with periocular nodular lesions. Histopathology and immunohistochemistry confirmed granulomatous dermatitis with intrahistiocytic* Leishmania* amastigotes. Additionally, *Leishmania infantum* DNA was detected in paraffin embedded skin biopsy. Following surgical removal of the lesions, sequential serological monitoring using enzyme-linked immunosorbent assay, and immunofluorescent antibody tests were performed over 90 days. Enzyme-linked immunosorbent assay and Western Blot results varied depending on conjugate type (Protein A versus Protein A/G). Western Blot revealed immunoreactivity against multiple *Leishmania infantum* antigen fractions, including early infection markers 14–16 kDa by conjugated Protein A/G and the absence of band by conjugated Protein A. Immunofluorescent antibody test using an anti-horse IgG fluorescein-labeled conjugate, where titers declined from 1:160 at 45 days to seronegativity by day 90, indicating antibody seroreversion within three months post-surgery. Similar results were detected by enzyme-linked immunosorbent assay with the absence of detection by Protein A but seropositivity was detected by Protein A/G. Finally, no parasitemia was detected by molecular test during the follow-up. This case represents the first documented seroreversion kinetics in equine leishmaniosis and highlights the low and transient humoral response in horses compared to dogs. Our findings underscore the importance of combining histopathology and immunohistochemistry, for the accurate equine leishmaniosis diagnosis, and emphasize the need for further studies to clarify the epidemiological role of horses in *Leishmania infantum* transmission.

## Background

Leishmaniosis is a zoonotic, vector-borne disease caused by the protozoan parasite *Leishmania infantum* and transmitted by infected sand flies across European Mediterranean countries. From an epidemiological standpoint, dogs are the main reservoir in the domestic transmission cycle of human leishmaniosis (Quinnell and Courtenay 2009). However, the presence of infection has also recently been detected in other domestic species, such as pet ferrets (Giner et al. 2020) and livestock (Cardoso et al. 2021).

So far, research has focused on the epidemiological role of dogs and cats as domestic hosts within the domestic transmission cycle, as well as on a variety of wild species participating in the sylvatic cycle of the infection (Millán et al. 2014). More recently, seropositivity has been reported in livestock, mainly clinically healthy sheep, goats, cattle, and pigs, in the southeast Iberian Peninsula (Martín-Sánchez et al. 2025). Furthermore, seropositive sheep has also been detected in the northern Iberian Peninsula, with a reported seroprevalence of 9.27% (Villanueva-Saz et al. 2024a). A clinical case of leishmaniosis in a goat has also been described in Spain, characterized by exfoliative dermatitis and lymphadenomegaly. In that case, moderate levels of anti-*Leishmania* antibodies were detected using an in-house enzyme-linked immunosorbent assay (ELISA), and treatment with meglumine antimoniate combined with allopurinol led to complete clinical recovery (Ruiz et al. 2023).

In recent years, studies assessing the role of other livestock species, particularly horses, in the epidemiology of *L. infantum* in Europe have become increasingly common. The first case of *Leishmania* infection in a horse in Europe was reported in 1996 (Solano-Gallego et al. 2003), and since then, the number of naturally occurring cases of equine leishmaniosis (EL) has continued to rise, not only in Spain but also worldwide. However, detailed clinical descriptions of the disease remains limited. The most frequently reported clinical manifestations of EL are dermatological, consisting mainly of single or multiple papules, papulonodules, or nodules, some of which may ulcerate. These lesions are most frequently located on the face and head, although they may also appear on axillae, groin, inguinal region or metatarsus, areas where female sand flies typically feed (Table 1). In contrast, systemic clinical signs and significant clinicopathological alteration are rarely reported in case studies of EL.

**Table 1:** Results of case reports of equine leishmaniosis by *L. infantum* in Europe

Publication year	Country	Animal characteristics	General signs	Dermatological lesions	Clinicopathological findings	Dermatological resolution	Confirmatory Techniques for *Leishmania infantum* Infection						Reference
							Serological methods	Molecular methods	Histopathology	Immunohistochemistry	Culture	Other	
2002	Germany	3.5-year-old Bavarian colt detected in 2000	Absence of general signs	Multiple small nodular dermal lesions were located on the right lower eyelid and in the temporal region lateral to the right eye	Not described	Nodules disappeared spontaneously within few months	IFAT and ELISA but anti-*Leishmania* antibodies ere not detected	Yes, positive result (PCR and restriction fragment length polymorphism (RFLP)	Yes (granulomatous Inflammation with the presence macrophages contained numerous ovoid amastigote protozoan organism)	Yes, positive result	No	Yes, positive result by Transmission electron microscopy (presence of several amastigotes within parasitophorous vacuoles in macrophages)	Koehler et al. 2002
2002	Spain	Not described	Not described	Papulonodular lesions on the head, axillae and groin	Not described	Not described	Not described	Not described	Not described	Not described	Yes, positive isolation	Not described	Portús M et al. 2002
2003	Spain	Case 1: A 3—year-old male Andalusian horse detected in 1996 Case 2: A 10-year-old female mixed breed horse detected in 1999 Case 3: A 3. 5—yea r-old female Andalusian horse detected in 2000	Absence of general signs	Case 1: cutaneous papulonodular lesions on the head Case 2: Several cutaneous nodules on the inguinal area Case 3: papules and ulcerated nodules on the head, axillae and groin	Not described	Case 1: The lesions healed spontaneously after 5 months Case 2: The lesions healed spontaneously after 4 months Case 3: The lesions healed spontaneously after 3 months	ELISA (case 3)		Yes, Nodular to diffuse granulomatous dermatitis with scattered multinucleated giant cells among the cellular infiltrates, accompanied by an intense lymphocytic infiltrate (All cases)	Yes, positive result (All cases)	Yes, positive isolation (case 3)	Yes, Lymphocyte proliferation assay (LPA) in case 3 with a strong lymphocyte proliferative response	Solano-Gallego et al. 2003
2005	Portugal	17-year-old male mixed-breed (Anglo-lusitano) horse	Absence of general signs	Single irregular ulcerative skin lesion in the right metatarsus	Not described	The lesion healed spontaneously and relapsed after three months	Presence of anti-*Leishmania* antibodies detected by counterimmunoelectrophoresis (CIE)	Yes, positive result by real time PCR	Not described	Not described	Not described	Not described	Rolão et al. 2005
2014	Portugal	2-year-old male Belgisch Warmbloed Paard	Absence of general signs	Ulcerated nodular lesion in the left face	Not described	No recurrence after surgical removal	Presence of anti-*Leishmania* antibodies detected by direct agglutination test (DAT)	Yes positive result. DNA sequence analysis confirmed *L. infantum*	Yes, Nodular to diffuse periadnexal inflammation with macrophages, few lymphocytes, plasma cells, and multinucleated giant cells, associated with ulceration. Amastigote-like organisms observed in macrophages	Yes, positive result	Not described	Not described	Gama et al. 2014

Reliable diagnosis of EL generally requires a combination of confirmatory techniques. In clinical studies, histopathological examination supported by specific immunohistochemistry, together with serological and molecular methods, is commonly employed. Serological techniques most frequently used for detecting anti-*Leishmania* antibodies include the immunofluorescent antibody test (IFAT) (Koehler et al. 2002), direct agglutination test (DAT) (Gama et al. 2014), ELISA (Solano-Gallego et al. 2003), and counterimmunoelectrophoresis (CIE) (Rolão et al. 2005). In contrast, other assays as Western Blot (WB) have not yet been described in either clinical or seroepidemiological studies of EL.

Given the scarce clinical data on the humoral response in naturally acquired *L. infantum* infection in horses, the aims of this study were: (1) to assess the serological evolution of antibodies against *L infantum* following surgical excision of dermatological lesions; and (2) to characterize the immunoreactivity pattern using WB.

## Case presentation

**Fig. 1:**
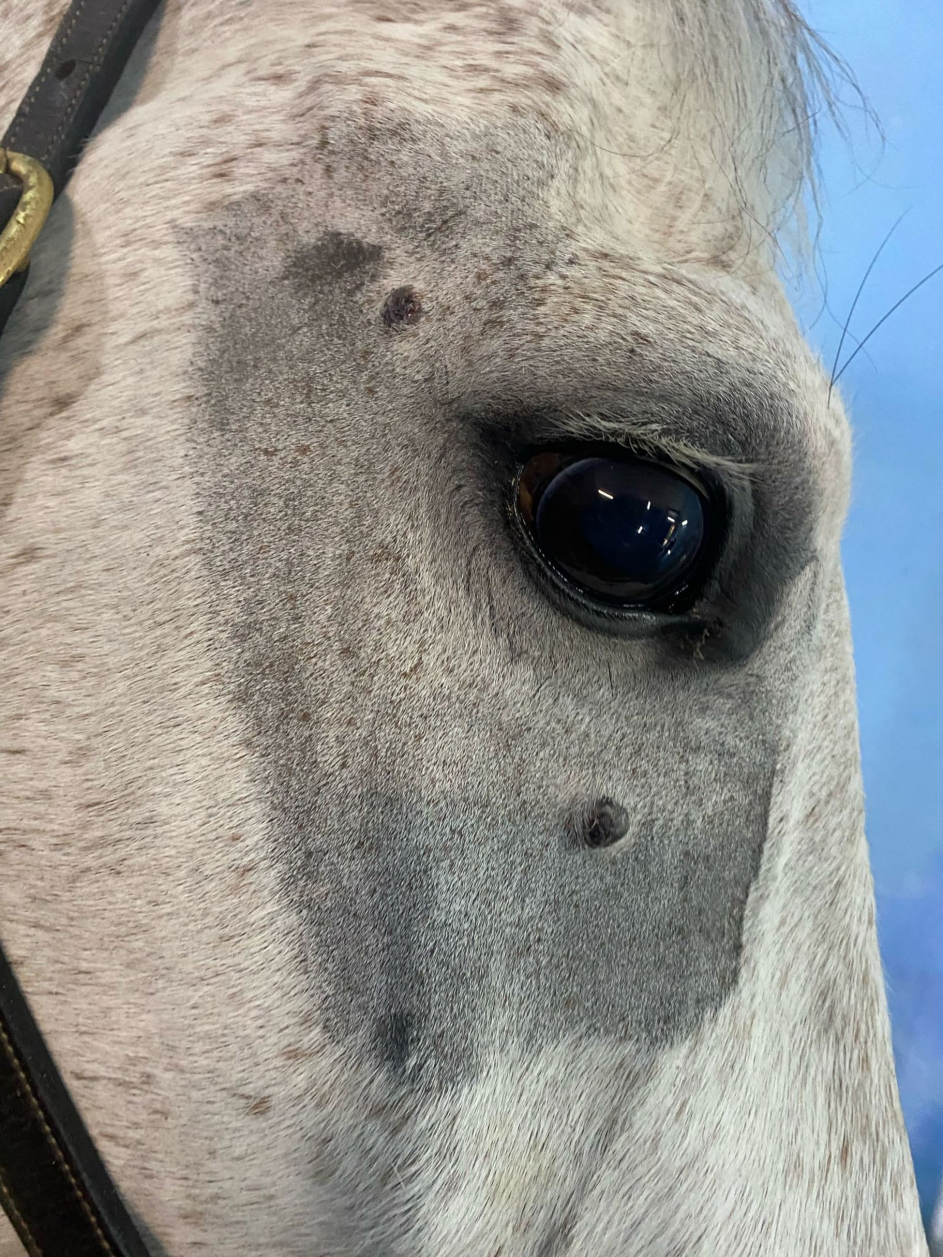
Presence of two periocular nodular masses, each measuring approximately 1 × 1 cm, located on the right side of the face, one supraorbital and the other infraorbital

A 10-year-old grey mare of crossbred Central European descent from Mijas (Málaga, Spain) was examined by a local veterinarian for the presence of facial masses that had developed over a 3-month period. The horse was subsequently referred to a nearby veterinary hospital for surgical excision of two periocular nodular masses, each measuring approximately 1 × 1 cm, located on the right side of the face, one supraorbital and the other infraorbital (Fig. 1). A complete physical examination revealed no additional clinical abnormalities. Hematological and biochemical analyses were performed using an automated hematology analyzer (Procyte Dx®, Idexx, Westbrook, Maine, USA) and a chemistry analyzer (Catalyst One® Chemistry Analyzer, Idexx, Westbrook, Maine, USA), including measurements of glucose, creatinine, blood urea nitrogen (BUN), calcium, total protein, albumin, globulins, aspartate aminotransferase (AST), alkaline phosphatase (ALP), gamma-glutamyl transferase (GGT), lactate dehydrogenase (LDH), Creatine kinase (CK), and bilirubin. No clinicopathological abnormalities were detected. The lesions were excised under local anesthesia and sedation, and the surgical wounds were closed with staples. The masses were fixed in 10% neutral-buffered formalin, paraffin-embedded, and sectioned at 4 µm for hematoxylin and eosin (H&E) staining. immunohistochemistry (IHC) was also performed to detect *Leishmania* spp. antigens, following a previously-described protocol (Giner et al. 2020).

**Fig. 2:**
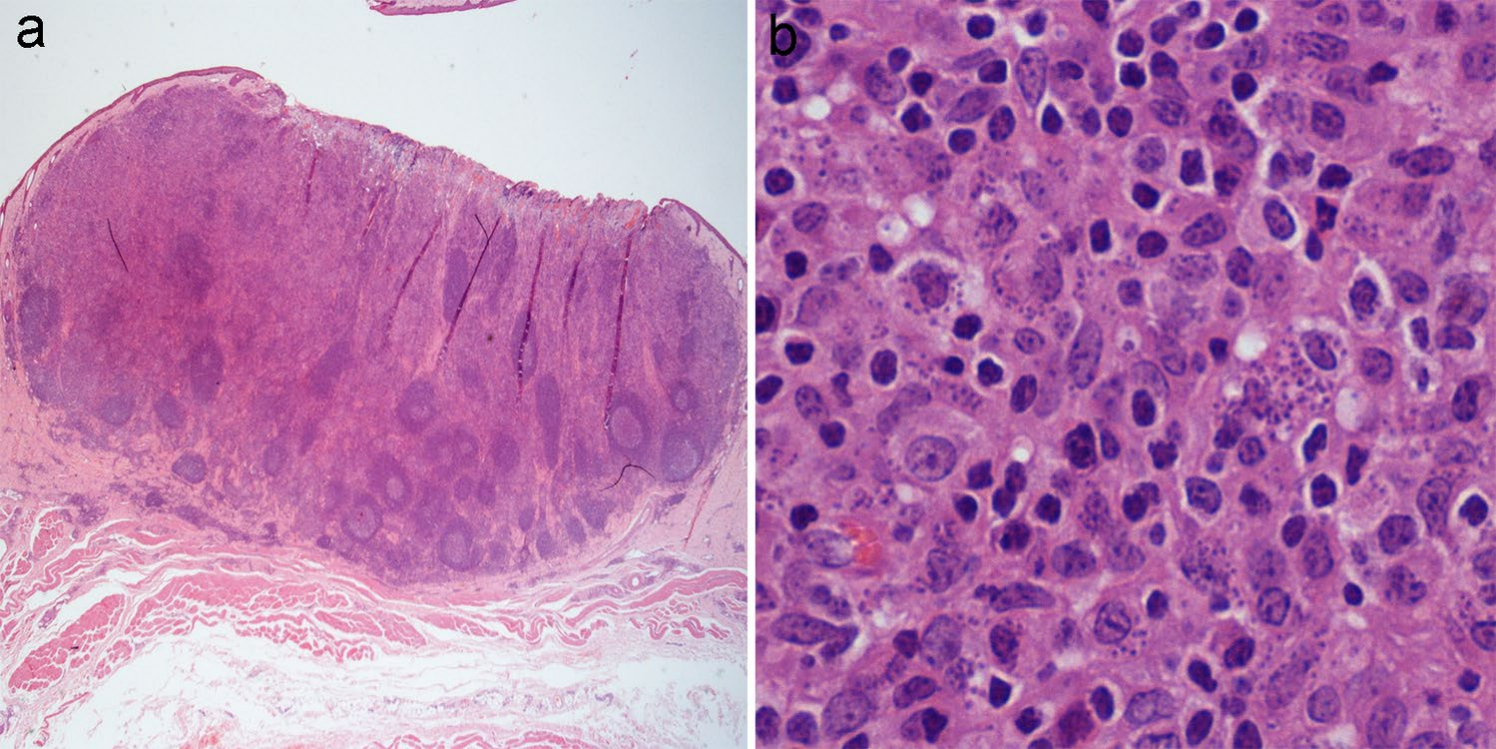
Microscopic lesions in the equine case report with cutaneous leishmaniosis caused by *Leishmania infantum*. Hematoxylin–eosin (HE). **a**) Skin. Dermal inflammatory nodule in the superficial dermis showing ulceration of the epidermis, high cellular density and conspicuous tertiary lymphoid follicles. × 1. **b**) Skin. Intracytoplasmic protozoal organisms compatible with *Leishmania spp*. amastigotes in macrophages. Hematoxylin–Eosin, × 60

**Fig. 3:**
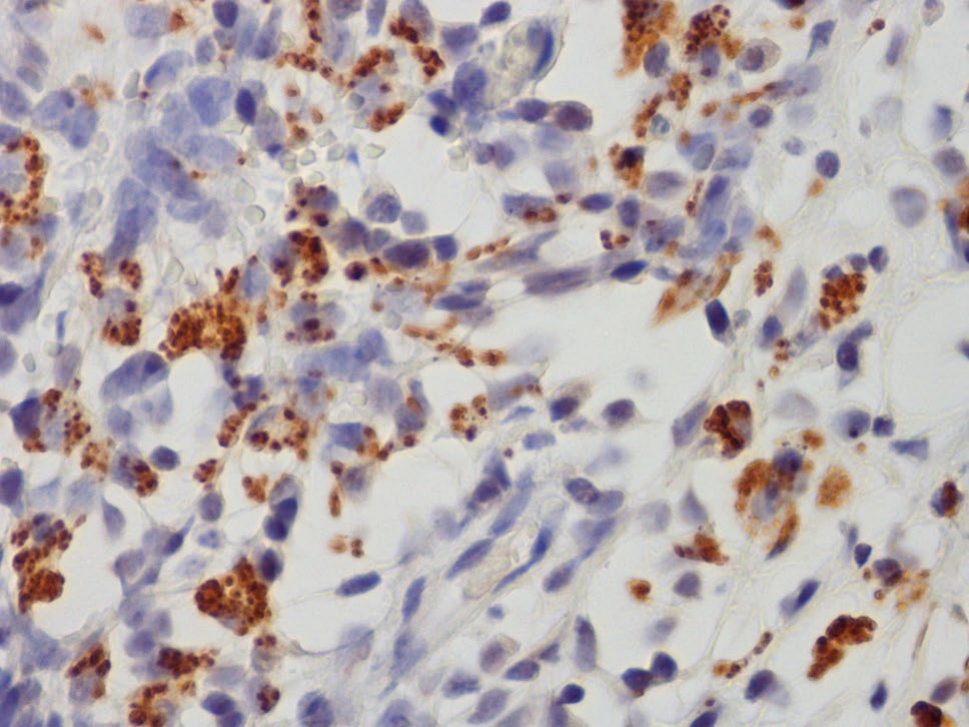
Skin. Intracytoplasmic protozoal organisms are positive by immunohistochemistry against *Leishmania spp*. IHC × 60

Histopathological examination of both masses revealed identical lesions. In the superficial dermis, there was a well-demarcated inflammatory nodule containing tertiary lymphoid follicles with active germinal centers (Fig. 2a). This nodule was predominantly composed of macrophages and lymphocytes with fewer viable and degenerate neutrophils. Cells were arranged in cords, infiltrating dermal collagen bundles. The epidermis was multifocally to extensively ulcerated, with necrosis and serocellular crusts composed of fibrin, cellular debris, and both, viable and degenerated neutrophils. Numerous infiltrating macrophages exhibited a vacuolated cytoplasm containing multiple (1–8) intracytoplasmic protozoal organisms measuring 3–4 μm in diameter, round to oval in shape, each with a basophilic nucleus measuring 1–2 μm, morphologically consistent with *Leishmania spp.* amastigotes (Fig. 2b). Numerous oval microorganisms within cytoplasm of dermal macrophages exhibited strong positive immunolabeling by IHC (Fig. 3). The presence of *L. infantum* DNA in paraffin embedded skin biopsy was additionally evaluated by amplification of kinetoplast DNA sequence using a quantitative polymerase chain reaction (qPCR) (Francino et al. 2006), and positive result by PCR was obtained from this sample.

Following the diagnosis of EL, a serological follow-up was conducted using the most common serological methods, namely ELISA and IFAT, together with molecular detection of *Leishmania* spp. DNA from EDTA-treated blood samples. Serum and blood samples were collected every 45 days, when the horse tested seronegative. In addition, WB was included, despite its limited use in clinical EL, to characterize the inmmunoreactivity profile of anti-*Leishmania* antibodies. Serum protein electrophoresis was also carried out using agarose gel electrophoresis (AGE) as previously described (Villanueva-Saz et al. 2024b). Identical electrophoretic patterns were observed across all time points, with no evidence of pathological alterations.

For WB analysis, anti-*Leishmania* antibodies were detected using a whole *L. infantum* promastigote antigens, as previously described (Marteles et al. 2024). Horse sera were tested at a 1:100 dilution, employing two horseradish peroxidase-conjugated secondary reagents, Protein A and Protein A/G, each diluted 1:10,000. A sample was considered WB-positive when immunoreactivity against the 14 kDa and/or 16 kDa low-molecular-weight polypeptide fractions of the *L. infantum* antigen was observed. WB analysis revealed immunoreactivity against *Leishmania* antigen fractions of 14,16, 18, 24, 26, 28, 42, 44, 50, and 70 kDa at 45 days post-surgical removal when the Protein A/G conjugate was used, and no immunoreactivity when the Protein A conjugate was employed.

ELISA was performed as previously described (Marteles et al. 2024; Villanueva-Saz et al. 2024a). The working dilution of horse sera was 1:100, and two different conjugates, Protein A and Protein A/G conjugated to horseradish peroxidase (Thermo Fisher Scientific n.d.), were used at a 1:10,000 dilution. All sera and conjugates were diluted in phosphate-buffered saline containing 0.05% Tween 20 and 1% dry skimmed milk. Cut-off values were established at 0.260 and 0.300 optical density (OD) for Protein A and Protein A/G, respectively, calculated as the mean plus three standard deviations of values from 143 apparently healthy horses from a non-endemic area for *L. infantum* infection. Samples exceeding these cut-offs values were considered positive. In addition, two-fold serial dilutions (1:100 to 1:12,800) were tested on the same plate. Using the Protein A conjugate, the mare was seronegative at both 45 days (OD = 0.150) and 90 days (OD = 0.142) post-surgery. In contrast, with the Protein A/G conjugate, the mare remain weakly seropositive, showing low antibody levels (OD = 0.560 at 45 days; 0.290 at 90 days; 1:100 dilution). Serial dilution testing confirmed that OD values were below 0.260 for all samples with Protein A, whereas only a single positive result was observed at 1:200 dilution and 45 days with protein A/G (OD = 0.390), with all other dilutions below cut-off values.

An IFAT for detecting anti-*Leishmania*-specific immunoglobulin G (IgG) antibodies was carried out using an anti-horse IgG fluorescein-labeled conjugate, adapted from the feline protocol (Alcover et al. 2021). A cut-off titer ≥ 1:80 was used to define seropositivity. Serum samples were tested in two-fold serial dilutions to determine the end-point titer. Positive and negative controls were included on each slide. Results were evaluated independently by two trained researchers, with a third examiner resolving any dsicrepancies. The mare was seropositive at 45 days post-surgery (titer 1:160) and seronegative at 90 days (titer 1:40).

Finally, *Leishmania* spp. DNA detection in EDTA-blood samples was performed quantitative PCR (qPCR), as previously described (Villanueva-Saz et al. 2025a). The results were negative at both 45 and 90 days post-surgical removal of the lesions.

## Discussion and conclusions

In *Leishmania infantum* infection, serological diagnosis relies on detecting host antibody responses, whose magnitude and kinetics vary widely among species and individuals depending on age, immune status, and genetic background. Experimentally in dogs, antibodies typically appear within few weeks post-infection, reaching seropositive thresholds after about two months post-infection. Antibody titers continue to rise, peaking around three to six months after infection (median, 3 months) (Abranches et al. 1991; Martínez-Moreno et al. 1995; Olías-Molero et al. 2019). However, intervals for seroconversion in naturally infected dogs can range from 1 to 22 months (median, 5 months) (Alvar et al. 2004). More recently, in a cohort of dogs initially seronegative for leishmaniosis, 62.5% seroconverted during a one-year follow-up. Among these, 60% became seropositive within 3 months, 20% within 6 months, and the remaining 20% by 9 months post-infection (Alves et al. 2013). Limited information of seroconversion is available in EL due to both the scarce number of case reports published that evaluate the anti-*Leishmania* kinetics and the absence of commercial serological techniques that can be applied in a clinical setting. In this context, a horse from northern Portugal presenting with an ulcerated nodular lesion exhibited an initial DAT titre of < 25, which rose to 200 over a 13-month period following lesion onset, consistent with seroconversion (Gama et al. 2014).

In contrast to dogs, where elevated anti-*Leishmania* antibody levels are strongly correlated with a high parasite burden and, consequently, with the development of clinical disease (Reis et al. 2006). Conversely, low antibody levels in clinically normal dogs with a negative result on molecular and/or parasitological tests may indicate exposure or early stages of *Leishmania* infection (Paltrinieri et al. 2010).

Notably, recent evidence has also documented cases of clinical canine leishmaniosis without detectable humoral response, mainly presentations characterized by dermatological lesions (García et al. 2023) as well as nonspecific systemic signs (Villanueva-Saz et al. 2025b). In EL, most clinically affected cases exhibit low anti-*Leishmania* antibody levels when assessed by quantitative serological tests. Because serological methods alone may lack the sensitivity to detect subclinical infections, it is advisable to combine at least two diagnostic techniques of different nature for a more accurate detection of *Leishmania* spp. infection in such clinical scenarios. In this clinical case, the mare presented with nodular lesions, which are the most common clinical finding in affected horses, *Leishmania spp.* parasites were identified through histopathological examination using H&E staining in combination with specific IHC targeting *Leishmania.*

The low antibody titers observed in this mare are in alignment with those reported for cutaneous leishmaniasis (CL) in other species and help to explain the discrepancies often seen between clinical findings and serological results (Kar 1995; Goto and Lindoso 2010). In contrast to visceral leishmaniasis (VL), which is typically associated with a high systemic parasite burden and a marked humoral response (Pareyn et al. 2025), CL usually presents as a localized infection with a comparatively lower parasite load. This restricted distribution of parasites results in a weaker antigenic stimulus and, consequently, a less intense and sometimes barely detectable antibody response, which can reduce the sensitivity of serological assays in CL despite the presence of active infection (De Vries et al. 2015).

Immunologically, CL in humans (Castellano et al. 2009) and dogs (Solano-Gallego et al. 2011) is more strongly associated with a predominantly cell-mediated (Th1-type) immune response at the lesion site, whereas VL is classically linked to a more pronounced humoral (Th2-type) response. By analogy, a similar pattern is likely to occur in horses with cutaneous disease, where effective local cellular immunity may limit systemic spread of the parasite but also lead to relatively low circulating antibody levels. This could account for the modest titers and even negative results obtained with some of the serological methods employed in our case, despite clear clinicopathological evidence of *Leishmania* infection within the cutaneous granuloma.

Further differences between CL and VL also affect the specificity and clinical utility of serological tests. VL in a given region is often associated with one predominant *Leishmania* species, which can favor higher test specificity, whereas CL may be caused by several dermotropic species, increasing the potential for cross-reactivity and variable test performance (Gow et al. 2022). In addition, in VL, antibody-based tests are often used not only for diagnosis but also, with caution, for monitoring treatment response and detecting relapses, since antibody titers tend to remain high for prolonged periods after infection. In CL, however, antibody levels are generally lower and may decline more rapidly following lesion resolution, limiting the usefulness of serology for follow-up (Reimão et al. 2020). Taken together, these aspects support that the low antibody titers found in this mare are compatible with a cutaneous form of leishmaniasis and underscore the need to interpret serological findings in equine CL in conjunction with direct (parasitological or molecular) evidence found in samples from lesions.

Although the first serum sample from this patient was not collected at the same time as the skin biopsy, seroreversion kinetics were subsequently analysed using ELISA and IFAT. In addition, no previous information on the kinetics of antibody seroreversion in horses with *L. infantum* infection has been published to date. To the best of our knowledge, this is the first report demonstrating that *L. infantum* infection in horses is followed by a decrease in anti-*Leishmania* antibody levels within 3 months after lesion removal, whereas in dogs, seroreversion following anti-*Leishmania* treatment has been documented, with anti-*Leishmania* antibody levels typically showing a progressive decline within the first 6–12 months post-therapy treatment (Amusategui et al. 1998; Solano-Gallego et al. 2011).

One of the main challenges in assessing seroreversion in horses with EL is the small number of reported cases, often presenting without additional clinical signs or laboratory abnormalities suggestive of the disease. This, combined with the lack of commercially validated diagnostic tests for use in horses, makes it difficult to reliably evaluate humoral immune kinetics.

The detection of low anti-*Leishmania* antibody levels in horses with EL may be attributed to the immunoprofile typically developed in this species, characterized by a strong *Leishmania*-specific cellular response without developing clinical symptoms, as detected by lymphocyte proliferation assay (Solano-Gallego et al. 2003; Fernández-Bellon et al. 2006). It is also likely that subclinical infections are more common in horses than the clinical forms observed in dogs.

In this mare, ELISA and IFAT tests were used to evaluate the kinetics of anti-*Leishmania* antibodies after surgical removal of the skin lesion. In the ELISAs based on Protein A and Protein A/G conjugates, differences in the detection of seropositive samples were observed, following the dilution instructions provided with each conjugate. By contrast, a study evaluating the humoral response to *L. infantum* in healthy horses in Spain used two different ELISA conjugates: Protein A (Sigma-Aldrich) and rabbit anti-horse IgG (Sigma-Aldrich Chemie). The assays differed in serum dilution (1:100 for the Protein A-ELISA and 1:200 for the anti-horse IgG-ELISA) and conjugate dilution (1 mg/mL for the Protein A conjugate and 1:4000 for the anti-horse IgG-ELISA), but used the same antigen type and concentration as the ELISAs in the present study. That study detected seropositive animals only when the ELISA was performed with Protein A, whereas no positive results were obtained with the anti-horse IgG-ELISA (Fernández-Bellon et al. 2006). Based on our results with serum serial dilutions, the absence of positive results in the anti-horse IgG-ELISA was likely due to a serum dilution-associated effect rather than to a lack of binding between the conjugate and immunoglobulin G.

Additionally, discrepancies in the results obtained with Protein A in our study, particularly the lack of positive ELISA results, compared with those from a study evaluating the humoral response to *L. infantum* in healthy horses in Spain (Fernández-Bellon et al. 2006), highlight the importance of understanding the binding strength between conjugates and species-specific antibodies. Protein A demonstrates variable affinity across species, with limited binding to total IgG in horses, as reported in a technical document (Thermo Fisher Scientific 2013) and confirmed in other studies (Italiya et al. 2025). In our case, both the ELISA and the WB that used Protein A failed to detect optical density values above the cut-off or to identify an immunoreactivity band pattern, respectively.

WB analysis revealed immunoreactivity against multiple *Leishmania* antigen fractions, indicative of a pleomorphic response. Specific antibodies against low-molecular weight proteins (14 and/or 16 kDa) are considered indicative of a positive result, as these bands are the first to appear and therefore represent an early marker of infection (Mary et al. 1992), described in the present case.

In this patient, detection of the parasite in the affected skin confirmed the cause-effect relationship (Maia and Campino 2008), and the presence of low anti-*Leishmania* antibody levels was attributed to the disease rather than serological exposure. In endemic areas, seasonal variation in antibody levels has been reported in dogs (Cavalera et al. 2021), domestic ferrets (*Mustela putorius furo*) (Villanueva-Saz et al. 2021), European mink (*Mustela lutreola*) (Del Carmen Aranda et al. 2025) and horses (Sgorbini et al. 2014) after natural exposure during both the sand fly transmission and non-transmission periods. This variation suggests a transient humoral response to *L. infantum*, as both infective sand fly bites, involving inoculation of mature metacyclic promastigotes, and non-infective bites, involving inoculation of immature promastigotes, can induce *Leishmania*-specific antibodies (Gradoni et al. 2019).

In conclusion, this report documents the first characterization of anti-*Leishmania* antibody seroreversion in a horse after the surgical excision of cutaneous granulomas, revealing low humoral response occurs despite confirmed infection. These findings highlight the need to combine multiple confirmatory diagnostic techniques for the diagnosis of EL and support targeted surveillance in endemic areas to better define the role of horses in the epidemiology of *L. infantum*.

## Data Availability

Not applicable.
